# Ticks and associated pathogens recovered from dogs and cats during a longitudinal collection study at veterinary practices in France

**DOI:** 10.1186/s13071-026-07249-9

**Published:** 2026-03-09

**Authors:** Christophe Marques Alves, Juliette Lavarec, Albert Agoulon, Franck Guetta, Céline Hubinois, Guillaume Queney, Jacques Guillot

**Affiliations:** 1 MSD Animal Health, 49071 Beaucouzé Cedex, France; 2 Antagene, Animal Genomics Laboratory, 69890 La Tour-de-Salvagny, France; 3 Oniris, INRAE, BIOEPAR, 44300 Nantes, France; 4 Antech Diagnostics, Mars Petcare Science Diagnostics, 91300 Massy, France; 5 Oniris, Dermatology-Parasitology-Mycology, 44300 Nantes, France; 6 Univ Angers, Univ Brest, IRF, SFR ICAT, 49000 Angers, France

**Keywords:** Ticks, Dogs, Cats, France, Tick-borne pathogens, Endosymbionts

## Abstract

**Background:**

Documented changes in spatial and seasonal tick distribution highlight the importance of continuous surveillance. This paper documents results of a year-round sampling campaign in France, including identification of ticks and associated pathogens recovered from dogs and cats, as part of the European project “Protect Our Future Too.”

**Methods:**

Ticks were collected from dogs and cats presented to 35 veterinary practices from 27 administrative French departments between April 2021 and July 2022. DNA extracted from each tick sample was amplified by polymerase chain reaction (PCR) for simultaneous detection of 18 types of protozoan or bacterial microorganisms.

**Results:**

Among 777 collected ticks, 6 species were morphologically identified in descending prevalence order as: *Ixodes ricinus* (58.3%), *Dermacentor reticulatus* (24.2%, mainly on dogs), *I. hexagonus* (7.2%, mainly on cats), *Rhipicephalus sanguineus* sensu lato (3.6%, mainly on dogs), *I. canisuga* (one tick collected from a cat), and *Haemaphysalis punctata* (one tick from a dog). Geographical distribution varied by tick species: *I. ricinus* and *D. reticulatus* were more frequent in northeast France, whereas *R. sanguineus* (s.l.) was predominant in southeast France. Ticks were collected throughout the study period but peaked in spring and early summer for *I. ricinus* and late winter and spring for *D. reticulatus*. The ticks *R. sanguineus* (s.l.) were collected only during summer. In total, 71.0% of the ticks were positive for DNA of at least one microorganism. *Anaplasma* bacteria were most frequent (up to 75.3% in *I. ricinus*) followed by *Rickettsia* (up to 49.5% in *D. reticulatus*). Piroplasm DNA (*Babesia*/*Theileria*/*Cytauxzoon* spp.) was found in 6.4% of *I*. *ricinus*, 5.3% of *D. reticulatus*, and 3.6% of both *I. hexagonus* and *R. sanguineus* (s.l). *Borrelia burgdorferi* sensu lato DNA was detected in 10.2% of *I. ricinus*. *Mycoplasma haemominutum/haematoparvum* DNA was detected in 21.8% of *D. reticulatus*, whereas *M. turicensis* DNA was detected in 14.3% of *I. hexagonus.*

**Conclusions:**

Results of this study show that ticks are a year-round risk for dogs and cats in France, and tick-borne pathogens are present as mono- or coinfections at high frequencies. Tick control recommendations for veterinarians and dog and cat owners should incorporate these risks.

**Graphical abstract:**

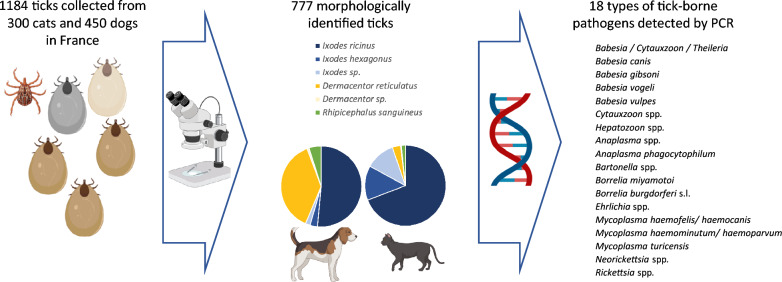

## Background

Ticks are common ectoparasites of dogs and cats worldwide. They are directly pathogenic through blood ingestion and transmit pathogens that impair animal health and can be zoonotic. The most commonly detected European ticks of dogs and cats are *Ixodes ricinus*, *I. hexagonus*, *Dermacentor reticulatus*, and *Rhipicephalus sanguineus* sensu lato [1]. These ticks do not have a uniform distribution and their range has changed over recent decades with a northward and higher elevation shift, particularly for *I. ricinus* [2–4]. Concomitantly, *D. reticulatus* ticks have expanded their range in central Europe [5, 6]. The brown dog tick, *R. sanguineus* (s.l.), traditionally associated with warm and dry Mediterranean basin ecosystems, is increasingly found, along with its transmitted pathogens, in temperate European regions, including the United Kingdom (UK) [7, 8]. Several factors are likely responsible for these changes. Deforestation and decreased agricultural diversity may contribute to *D. reticulatus* establishment in many regions [9], and increased temperatures facilitate lifecycle completion within 1 year. Recent mild winters in western and central Europe favor year-round *I*. *ricinus* and *D*. *reticulatus* activity [10–12]. Increases in average April to September temperatures favor *R. sanguineus* (s.l.) population spread into previously tick-free areas, including temperate regions, beyond the Mediterranean basin [13]. These tick activity period changes in Europe are also increasing pathogen transmission risks.

Several nationwide surveys were conducted in Europe to identify tick species infesting dogs and cats, including in Belgium [14], the UK [15, 16], Spain [17], Greece [18], Denmark [19], Latvia [20], the Netherlands [21], Germany and Austria [22], and Finland [23]. These studies usually include detection of bacterial and protozoan pathogens within collected ticks. A large tick surveillance program has not been completed in companion animals in France and distribution data for common tick species and their associated pathogens are limited. A small survey In 2001 and 2002 among 12 veterinary practices in northeast France [24] collected 181 ticks from 100 dogs and 22 cats. The tick *I. ricinus* was the dominant species (81.4% of ticks from dogs and 97.2% from cats). The complex species *R. sanguineus* (s.l.) was identified in 3.4% of ticks from dogs and 2.8% from cats, while *D. reticulatus* was found only on dogs (15.2% of ticks). Examination of companion animals living in five European countries, including France, resulted in submission of 140 tick samples from 8 French veterinary practices [1]. These were identified as *I. ricinus* (28 ticks from dogs and 24 from cats), *I. hexagonus* (5 ticks from dogs and 5 from cats), *D. reticulatus* (25 ticks only from dogs), and *R. sanguineus* (s.l.) (37 ticks from dogs and 16 from cats).

To fill the gap in knowledge in France, this study aimed to investigate the distribution of hard ticks and associated pathogens in companion dogs and cats. The survey, carried out over 1 year and based on ticks collected in veterinary practices in different French regions, is a component of the European project “Protect Our Future Too” to investigate effects of climate change on dog and cat health and behavior.

## Methods

### Sample collection

A total of 35 veterinary practices across France were recruited between the end of 2020 and early 2021 to collect ticks from dogs and cats presented to the practice with a naturally acquired infestation. Each practice received a tick collection kit including tick removal tools, tick collection tubes filled with absolute ethanol (one tube per animal), and a questionnaire. Dog and cat owner locations were not recorded for data protection, therefore tick locations were assigned to the veterinary practice where the ticks were collected. Questionnaire responses were matched to tick collection tubes using a unique identification number and animal travel history over the prior 2 weeks was documented. Collected ticks stored in alcohol tubes were sent every 2 months to the parasitology department of the Veterinary College of Nantes (Oniris), France. Ticks were morphologically identified using a binocular microscope and conventional descriptions and keys [25, 26]. All tubes were forwarded to a diagnostic service (Antagene, Lyon, France) for protozoan and bacterial microorganisms detection.

### DNA extraction

Ticks were sorted by size: small (< 1 mm), medium (1–4 mm), and large (> 4 mm). If there were multiple ticks from one species in a tube, one tick was selected for testing. Large and medium ticks were cut lengthwise. One half was fixed in absolute ethanol at −20 °C for further analysis if needed. The other half of large and medium ticks, and all small ticks, were diced and placed in a tube filled with 1X PBS and glass beads. All tick pieces were vibro-ground (MM 400, Retsch) for 5 min at 30 Hz then lysed with proteinase K and incubated 1 h at 70 °C. An internal positive control (IPC) consisting of a synthetic plasmid comprising DNA from alga, cocoa, and eucalyptus was added to each lysate before extraction to verify extracted DNA was of sufficient quantity and quality, and to detect DNA inhibitors. Two negative controls were also added to each extraction plate. DNA was extracted (KingFisher Flex instrument, Thermo Fisher Scientific) using magnetic beads (ID Gene® Mag Universal Extraction kit, ID.vet Genetics).

### PCR assays

The PCR assay allowed for simultaneous detection of 18 types of protozoan and bacterial microorganisms potentially involved in vectored diseases of cats and dogs: *Theileria* spp., *Babesia* spp., *B. canis*, *B. gibsoni*, *B. vogeli*, *B. vulpes*, *Cytauxzoon* spp., *Hepatozoon* spp., *Anaplasma* spp., *Anaplasma phagocytophilum*, *Bartonella* spp., relapsing fever group *Borrelia* spp., *Borrelia burgdorferi* sensu lato (s.l.), *Ehrlichia* spp., *Mycoplasma haemofelis/haemocanis, Mycoplasma haemominutum/haematoparvum, Mycoplasma turicensis, Neorickettsia*, and *Rickettsia* spp.

Primers allowed the species or genera specific amplification of each pathogen. For bacteria, genes targeted were: 16S ribosomal RNA, *flagellin B*, and *citrate synthase* and for protozoa, the 18S gene was targeted. The primer sequences used in this study are proprietary and therefore cannot be disclosed.

For *Theileria/Babesia*, a first primer set amplified all species belonging to these genera, then four other primer pairs specifically amplified the main species involved in European cat and dog vectored diseases: *Babesia canis*, *Babesia gibsoni*, *Babesia vogeli*, and *Babesia vulpes*. The same design was also made for *Anaplasma*: a first generic primer set amplified all the species within the genus, then a more specific primer set amplified only *A.* *phagocytophilum*.

The set of primers pairs was divided into three multiplex PCR. In each multiplex, a primer set targeting IPC was added.

The amplified fragment length polymorphism method (AFLP) was used to detect pathogens on the basis of PCR product size. Each primer set was labeled with either 6 carboxy-fluorescein (6-FAM) or photoinduced electron transfer (PET) fluorescent dye, and PCR products ranged from 100 to 300 pb. Every PCR product had a known size and a known dye, allowing for associated pathogen identification.

Duplicate external positive controls (EPC) were added to each PCR plate consisting of a synthetic plasmid composed of the target zone for each pathogen’s DNA. This verified that each primer pair was present in the multiplex and amplified the associated pathogen to preclude false-negative results. In addition to this EPC, two negative extraction controls were added to each plate, along with two negative PCR controls.

PCR reactions were performed in 96-well microplates in a 10 µl final volume containing 5 µl of Multiplex PCR Master Mix (Type-It Microsatellite Pcr Kit, Qiagen), and 3 µL of DNA template. The PCR cycling protocol consisted of 95 °C for 5 min, followed by five touchdown cycles of 95 °C for 30 s, 62 °C to 58 °C for 90 s (decreasing 1 °C per cycle), and 72 °C for 30 s, then followed by 39 cycles of 95 °C for 30 s, 58 °C for 90 s, and 72 °C for 30 s, ending with an extension of 72 °C for 10 min.

PCR products were resolved on a calibrated ABI PRISM 3130 XL capillary sequencer (ThermoFisher Scientific) under denaturing conditions (HiHi Formamide, ThermoFisher Scientific) with an internal size marker prepared once and dispatched equally in all sample wells of each marker run that guarantees the same calibration for all samples. Electropherograms were analyzed using GENEMAPPER 4.1 (ThermoFisher Scientific) and interpreted independently by two analysts. Negative extraction controls and negative PCR controls were checked first, as well as the EPC. A pathogen was considered positive when the peak at the expected size was greater than 2000 relative fluorescent units (RFU). The IPC presence was verified for each sample. If there was no IPC signal, but one or more pathogens was amplified, then the result was validated. On the contrary, if there was no IPC or pathogen signal, then the sample was re-run in PCR, with tenfold and 100-fold dilutions, to dilute potential PCR inhibitors. If there was still no amplification signal, the sample was considered to be not analyzable.

### Statistics

**Fig. 1:**
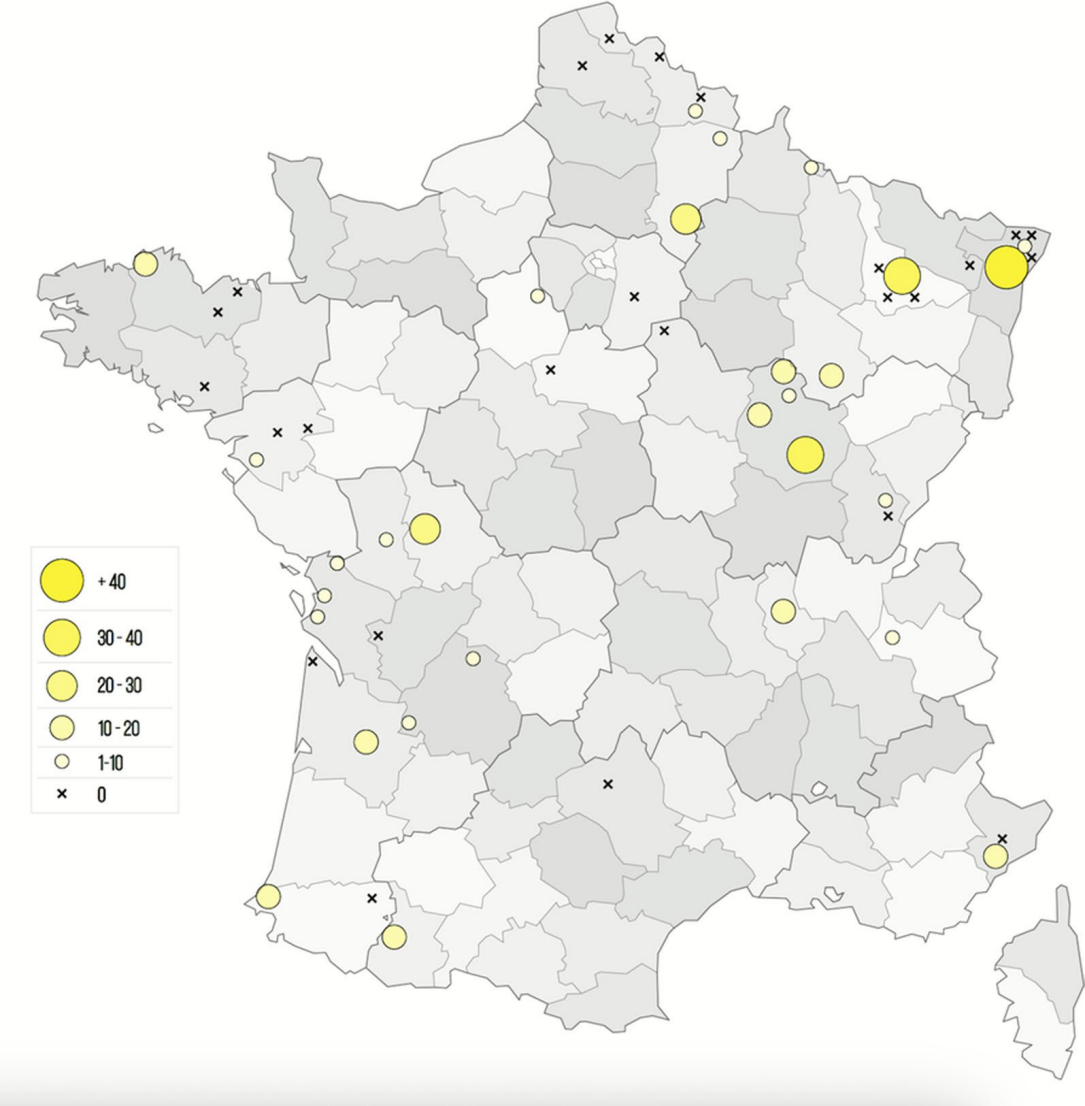
Location of the veterinary practices that accepted to participate to the study. Black crosses indicate the location of a clinic that did not collect ticks in the end. Circles indicate the location of a clinic that actively participated. The size of the circle is proportional to the number of ticks collected

Data analyses and cartography (except Fig. 1) were conducted in R (version 4.3.1) [27] using packages: *cartography*, *lubridate*, *maptools*, *shapefiles*, *sf*, *sp*, *tidyr*, and *tidyverse*. Categorical data were expressed as percentages and compared between groups using the Fisher’s exact test with *P* < 0.05 significant.

## Results

### Tick collection and identification

A total of 35 participating veterinary clinics collected 1184 ticks between April 2021 and July 2022 from 445 dogs and 300 cats (Fig. 1).

Six tick species were morphologically identified from four genera: *Ixodes ricinus* (58.3%), *Dermacentor reticulatus* (24.2%), *I. hexagonus* (7.2%), *Rhipicephalus sanguineus* (s.l.) (3.6%), *I. canisuga* (one tick on a cat), and *Haemaphysalis punctata* (one tick on a dog). Some ticks were identified to genus only: *Ixodes* sp. (5.8%) and *Dermacentor* sp. (0.6%). Species could not be identified because these were larvae or nymphs, or because the tick was damaged during tick removal.

Ticks identified on coinfested animals were most commonly *I. ricinus* and *D. reticulatus* (24 dogs and 2 cats). One cat was coinfested with three tick species (*I. ricinus*, *I. hexagonus*, and *D. reticulatus*).

**Fig. 2:**
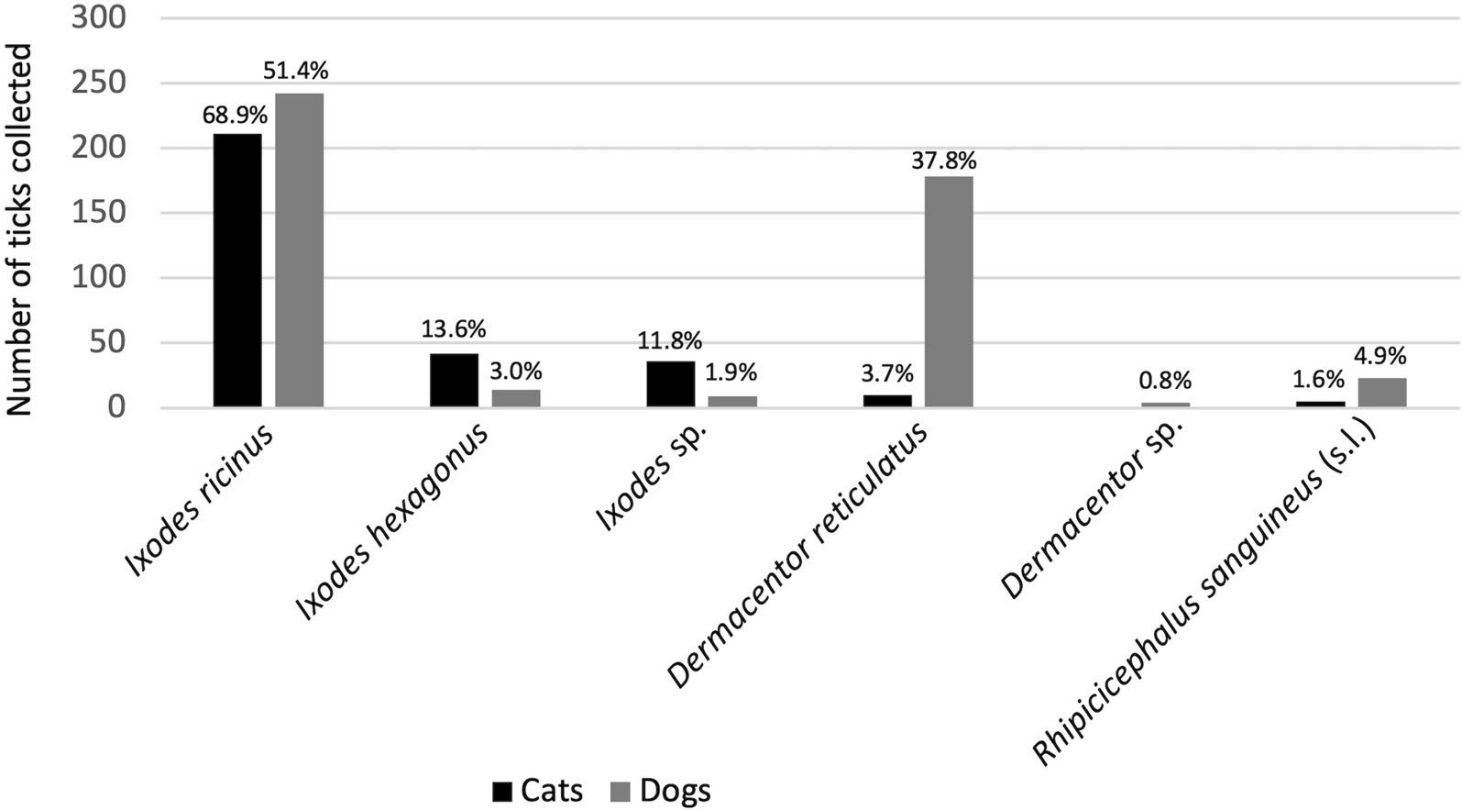
Species distribution of the most frequently encountered ticks from dogs and cats in France. Percentages correspond to the proportion for each animal host

Infesting tick species varied on dogs and cats (Fig. 2). Tick species collected from 445 dogs were composed of 242 (51.4%) *I. ricinus*, 178 (37.8%) *D. reticulatus*, and 23 (4.9%) *R. sanguineus* (s.l.) plus small numbers of *I. hexagonus* and *Hae. punctata*. Dogs were significantly more frequently infested by *D. reticulatus* (*P* < 0.001) or *R. sanguineus* (s.l.) (*P* = 0.045) than cats. Ticks collected from 300 cats were composed of 211 (68.9%) *I. ricinus* and 42 (13.6%) *I. hexagonus* plus small numbers of *D. reticulatus*, *R. sanguineus* (s.l.), and *I. canisuga*. Cats were significantly more frequently infested by *I. ricinus* (*P* < 0.001) or *I. hexagonus* (*P* < 0.001) than dogs.

**Fig. 3:**
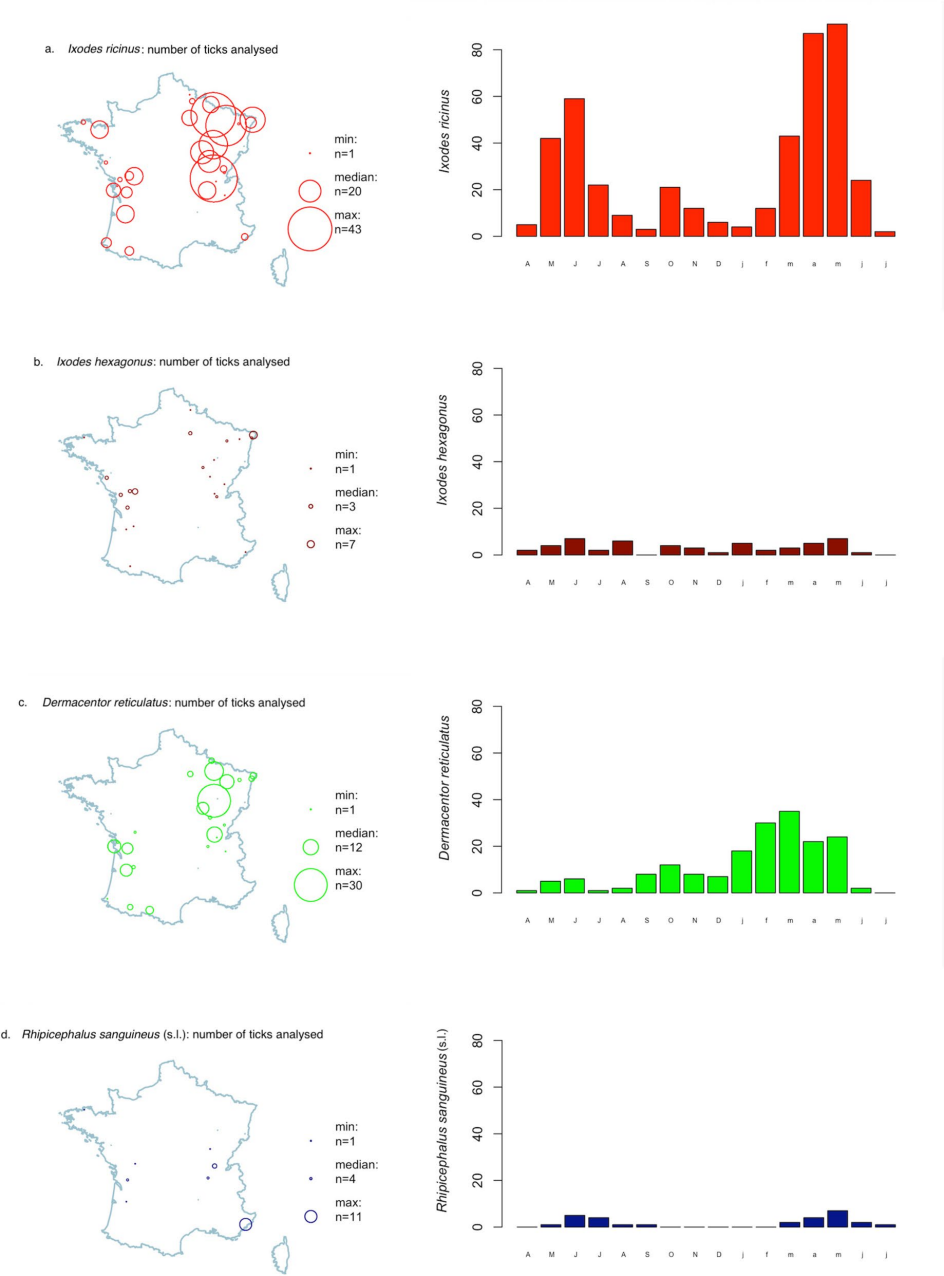
Geographic and temporal distribution of the most frequently encountered ticks from dogs and cats in France over the study period (April 2021–July 2022). **a**. *Ixodes ricinus*, **b**. *Ixodes hexagonus*, **c**. *Dermacentor reticulatus*, **d**. *Rhipicephalus sanguineus* (s.l.)

Geographic distribution of tick species varied: *I. ricinus* and *D. reticulatus* were more frequently collected in northeast France (Fig. 3a,c) whereas *R. sanguineus* (s.l.) was predominant in southeast France (Fig. 3d). No animal infested with *R. sanguineus* (s.l.) had a recent travel history.

Ticks were collected throughout the study period but tick infestation peaked in spring and early summer for *I. ricinus* (Fig. 3a) and late winter and spring for *D. reticulatus* (Fig. 3c). No peak was detected for *I. hexagonus* (Fig. 3b) and the few *R. sanguineus* (s.l.) ticks were collected only during summer (Fig. 3d).

For further analysis, one tick species specimen was randomly selected from individual animals with several ticks collected. As a consequence, a total number of 777 ticks were finally selected and examined for the presence of pathogens.

### Pathogen detection

Among the 777 ticks analyzed, 18 yielded no IPC or pathogen amplification, suggesting the presence of PCR inhibitors. Performing tenfold and 100-fold dilutions of the corresponding nucleic acid extracts restored amplification, confirming inhibition as the cause of the initial amplification failure and allowing results to be obtained.

PCR detected DNA of at least one microorganism in 552 (71.0%) of 777 ticks tested. The total frequency of any positive PCR was 82.3% in *I. ricinus*, 33.9% in *I. hexagonus*, 65.9% in *D. reticulatus*, and 57.1% in *R. sanguineus* (s.l.). Coinfection with DNA from more than one microorganism was found in 229 (29.5%) ticks with the highest prevalence at 46.8% for *D. reticulatus* (Table 1). The most frequent microorganisms were *Anaplasma* spp. bacteria (up to 75.3% in *I. ricinus*, 45.7% in *D. reticulatus* and 32.1% in *R. sanguineus* s.l.) and *Rickettsia* spp. (up to 49.5% in *D. reticulatus* and 35.7% in *R. sanguineus* s.l.) (Table 2). Excluding positive *Anaplasma* and *Rickettsia* spp. PCR results, the distribution of coinfections with pathogens was: 3 ticks (*I. ricinus*) positive for 3 pathogens and 21 ticks (14 *I. ricinus*, 1 *I. canisuga*, 2 *Ixodes* sp., and 4 *D. reticulatus*) positive for 2 pathogens. The most common coinfections were *Babesia*/*Theileria*/*Cytauxzoon* with either *Borrelia burgdorferi* (s.l.) or *Mycoplasma haemominutum*/*haematoparvum*.

**Table 1:** Results of global detection of pathogens in ticks

	*Ixodes ricinus*	*Ixodes hexagonus*	*Ixodes* sp.	*Dermacentor reticulatus*	*Dermacentor* sp.	*Rhipicephalus sanguineus* (s.l.)
Tick analyzed	453	56	45	188	5	28
Positive ticks for at least one pathogen	82.3%	33.9%	35.5%	65.9%	80.0%	57.1%
Positive ticks for one pathogen only	54.3%	26.8%	31.1%	19.1%	40%	32.2%
Positive ticks for two pathogens	20.7%	7.1%	4.4%	34.0%	40%	17.8%
Positive ticks for three pathogens	4.8%	–	–	11.7%	–	7.1%
Positive ticks for four pathogens	2.0%	–	–	1.1%	–	–
Positive ticks for five pathogens	0.2%	–	–	–	–	–

**Table 2:** Results of specific pathogen screening in ticks

	*Ixodes* *ricinus*	*Ixodes hexagonus*	*Ixodes* sp.	*Dermacentor reticulatus*	*Dermacentor* sp.	*Rhipicephalus sanguineus* (s.l.)
Ticks analyzed	453	56	45	188	5	28
*Babesia Cytauxzoon Theileria*	6.4%^a^ [4.3–9.1%]	3.6%^a^ [0.4–12.3%]	6.7%^a^ [1.4–18.3%]	5.3%^a^ [2.6–9.6%]	–	3.6%^a^ [0.1–18.3%]
*Babesia canis*	–	–	–	2.1% [0.6–5.4%]	–	–
*Babesia gibsoni*	–	–	–	–	–	–
*Babesia vogeli*	–	–	–	–	–	–
*Babesia vulpes*	0.4% [0.1–1.6%]	–	–	–	–	–
*Cytauxzoon* spp.	2.0%^a^ [0.9–3.7%]	–	4.4%^a^ [0.5–15.1%]	–	–	–
*Hepatozoon* spp.	4.4%^a^ [2.7–6.7%]	1.8%^a^ [0.0–9.6%]	–	1.1%^a^ [0.1–3.8%]	–	–
*Anaplasma* spp.	75.3%^a^ [71.0–79.2%]	1.8%^b^ [0.0–9.6%]	8.9%^bc^ [2.5–21.2%]	45.7%^d^ [38.5–53.2%]	40.0%^abcd^ [5.3–85.3%]	32.1%^cd^ [15.9%-52.4%]
*Anaplasma phagocytophilum*	1.5% [0.6–3.2%]	–	–	–	–	–
*Bartonella* spp.	–	1.8%^a^ [0.0–9.6%]	2.2%^a^ [0.1–11.8%]	–	–	–
*Borrelia miyamotoi*	2.4%^a^ [1.2–4.3%]	–	–	–	–	3.6%^a^ [0.1–18.3%]
*Borrelia burgdorferi* (s.l.)	10.2%^a^ [7.5–13.3%]	1.8%^a^ [0.0–9.6%]	–	–	–	3.6%^a^ [0.1–18.3%]
*Ehrlichia* spp.	0.2% [0.0–1.2%]	–	–	–	–	–
*Mycoplasma haemofelis/ haemocanis*	0.2% [0.0–1.2%]	–	–	–	–	–
*Mycoplasma haemominutum/ haematoparvum*	2.0%^a^ [0.9–3.7%]	–	–	21.8%^b^ [16.1–28.4%]	–	10.7%^ab^ [2.3–28.2%]
*Mycoplasma turicensis*	0.2%^a^ [0.0–1.2%]	14.3%^b^ [6.4–26.2%]	8.9%^bc^ [2.5–21.2%]	0.5%^ac^ [0.0–2.9%]	–	–
*Neorickettsia* spp.	–	–	–	–	–	–
*Rickettsia* spp.	16.1%^ab^ [12.8–19.8%]	16.1%^ab^ [7.6–28.3%]	6.7%^a^ [1.4–18.3%]	49.5%^c^ [42.1–56.8%]	80.0%^c^ [28.4–99.5%]	35.7%^bc^ [18.6–55.9%]

*Ixodes canisuga* (one specimen): positive PCR for *Babesia/Theileria/Cytauxzoon* and *Cytauxzoon*

*Haemaphysalis punctata* (one specimen): no positive PCR

Values within square brackets indicate the 95% confidence interval for prevalence

^a,b,c,d^for each pathogen, prevalences with different superscript letters are significantly different (*P*-value < 0.05)

**Fig. 4:**
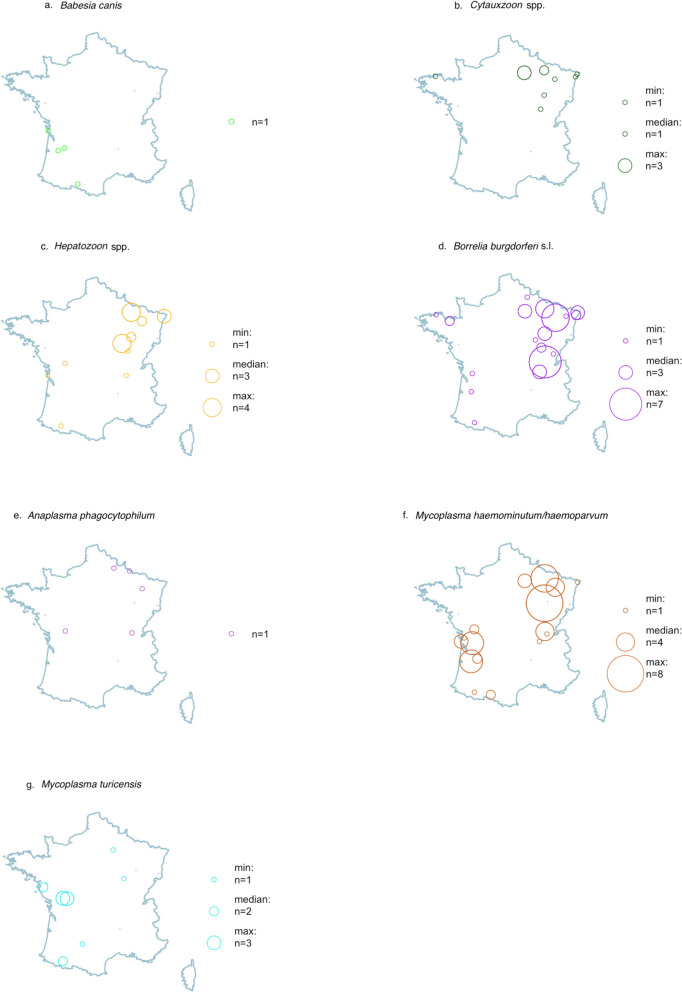
Geographic distribution of the ticks with positive PCR for the most important pathogens. **a**. *Babesia canis-*positive ticks, **b**. *Cytauxzoon-*positive ticks, **c**. *Hepatozoon-*positive ticks, **d**. *Borrelia burgdorferi* (s.l.)-positive ticks, **e**. *Anaplasma phagocytophilum*-positive ticks, **f**. *Mycoplasma haemominutum/haematoparvum*-positive ticks, **g**. *Mycoplasma turicensis*-positive ticks

Piroplasms (*Babesia*/*Theileria*/*Cytauxzoon* spp.) were found in 6.4% of *I. ricinus,* 3.6% of *I. hexagonus*, 5.3% of *D. reticulatus*, and 3.6% of *R. sanguineus* (s.l.). Specific PCR detected *B. canis* in 2.1% of *D. reticulatus* and *B. vulpes* in 0.4% of *I. ricinus*. The few *B.*. *canis*-positive ticks were collected only in southwest France (Fig. 4a). *Cytauxzoon* DNA was detected in 2.0% of *I. ricinus* and 4.4% of *Ixodes* sp. ticks. *Cytauxzoon*-positive ticks were collected almost exclusively from cats and only in northeast France (Fig. 4b).

No *B. vogeli* or *B. gibsoni* DNA was detected in any collected tick.

*Hepatozoon* DNA was detected in 4.4% of *I. ricinus*, 1.8% of *I. hexagonus*, and 1.1%, of *D. reticulatus* ticks. *Hepatozoon*-positive ticks were collected more frequently northeast France (Fig. 4c) and all collected *R. sanguineus* (s.l.) ticks were *Hepatozoon* spp. free.

*Borrelia burgdorferi* (s.l.) DNA was detected in 10.2% of *I*. *ricinus*, 1.8% of *I. hexagonus*, and 3.6% of *R. sanguineus* (s.l.), whereas *B. miyamotoi* DNA was detected in only 2.4% of *I. ricinus*, and 3.6% of *R. sanguineus* (s.l.) ticks. *Borrelia burgdorferi* s.l.-positive ticks were collected more frequently in eastern France (Fig. 4d).

*Mycoplasma haemominutum/haematoparvum* DNA was detected in 21.8% of *D. reticulatus*, 10.7% of *R. sanguineus* (s.l.), and 2.0% of *I. ricinus*, whereas *M. turicensis* DNA was detected mainly in *I. hexagonus* (14.3%). Other bacterial pathogens were not detected (*Neorickettsia* spp.) or rarely detected (*A. phagocytophilum*, *Bartonella* spp., *Ehrlichia* spp., and *M*. *haemofelis/haemocanis*). Four of five ticks positive for *A. phagocytophilum* were *I. ricinus* ticks in eastern France (Fig. 4e).

There were significant differences among tick species in their likelihood of testing positive for selected microorganisms. *Ixodes ricinus* ticks were more likely to test positive for *Anaplasma* spp. (*P* < 0.001), *B. burgdorferi* (*P* < 0.001), or *Hepatozoon* spp. (*P* = 0.032) compared with other tick species. *Ixodes hexagonus* ticks were more likely to test positive for *Mycoplasma turicensis* (*P* < 0.001), while *D. reticulatus* ticks were more likely to test positive for *Rickettsia* spp. (*P* < 0.001) or *B. canis* (*P* = 0.003).

## Discussion

The present study is the first to detect a high number of ticks infesting companion animals across France over more than a year of surveillance. It also provides original information about the prevalence of the most relevant pathogens in ticks. The main limitations of the study are inherent to veterinary-clinic-based sampling. The vast majority of collected ticks were adults, likely because smaller nymphs and larvae are difficult to detect in the fur of companion animals presented to veterinarians. There is a potential in-clinic PCR contamination and the clinical relevance of detecting DNA of pathogens in ticks remains unclear without corresponding host testing.

### Tick species and seasonal activity

The present study confirmed that *I. ricinus* is the most common tick infesting companion animals in France, while *I. hexagonus* is less common, although it is found particularly on cats. Previous investigations found consistent results in the Netherlands [28], Germany and Austria [22], the United Kingdom [15, 29], and Belgium [14]. Previous investigations also reported a significantly higher prevalence of *I. ricinus* on dogs compared with cats, whereas *I. hexagonus* was more often associated with cats [15, 16, 29]. These tick infestation differences between cats and dogs may result from behavioral differences between the host species, leading to tick exposure in distinct habitats. Outdoor cats may hunt small prey (frogs, small rodents, and birds) and consequently cohabitat with European hedgehogs, common *I. hexagonus* hosts [16, 30]. However, investigations from Germany [31], the Netherlands [28], and Belgium [14] found no significant difference in the distribution of *I. ricinus* and *I. hexagonus* between dogs and cats, suggesting that regional factors may also play a role. The third *Ixodes* species detected in the present study was *I. canisuga*, commonly known as the fox tick, and collected from one cat living in mountainous area of Savoy. In contrast, *I. canisuga* was frequently found on dogs in Ireland and the UK [29, 32].

Tick infestation peaked in spring and early summer for *I. ricinus.*

In accordance with previous investigations [32–36], our study demonstrated a prominent peak of *I. ricinus* infestation in spring and a smaller peak in autumn. This bimodal pattern corresponds to periods of moderate temperature and high humidity, with a higher spring activity compared with autumn, associated with diapause in decreasing day length conditions [37]. There can be year-to-year variation, with a single spring or summer peak reported for questing *I. ricinus* nymphs [38] and *I. ricinus* adults collected from dogs [31]. *Ixodes hexagonus* seasonality is less well described, with tick number fluctuations reported on hedgehogs [39] and dogs [31]. The tick *I*. *hexagonus* is an endophilic species and its seasonal fluctuations seem weaker compared with *I. ricinus*.

The species *D. reticulatus* was the second most frequently collected tick from dogs in the present study, accounting for one of four ticks (24.2%) collected over the whole study period and for the majority of ticks collected in January and February 2022. The tick *D. reticulatus* is traditionally associated with canine babesiosis foci in southwest France [40]. This tick is widely distributed across Europe and infestations on dogs, and to a lesser extent on cats, are increasingly reported in both endemic areas and areas previously considered nonendemic. In Germany, data from citizen-science investigations or ticks collected in veterinary clinics demonstrate that *D. reticulatus* has an expanded range, especially in the north [6, 22]. The tick *D. reticulatus* is established in parts of the Netherlands, Belgium, and the UK, with increasing numbers of infestations reported in dogs [14, 21, 28, 41]. This geographic expansion is likely driven by a combination of environmental changes, increased movement of dogs in Europe, and changes in land use to favor suitable habitats. The patchy and focal *D. reticulatus* distribution reflects its strong preferences for humid meadows, marshes, and forest edges; areas where dogs may frequently contact questing ticks. In accordance with previous investigations, our study confirmed that *D. reticulatus* ticks have a bimodal seasonal activity pattern. Adult ticks are primarily active in cooler months, with peak questing periods in late winter and early spring and again in autumn. The species *D. reticulatus* is well adapted to survive cooler temperatures and is often active when other tick species are dormant. Adult *D. reticulatus* can be observed questing at temperatures as low as 4–6 °C, providing adequate relative humidity [42].

The tick species *R. sanguineus* (s.l.) is primarily distributed in Mediterranean regions and was infrequently detected in the present survey. A limited number of veterinary clinics from southeast France participated in sample collection, which likely accounts for the low detection of *R. sanguineus* ticks (s.l.) in our study. Ticks were collected from some dogs residing outside the species’ typical geographical range and none of these dogs had a history of travel to Mediterranean countries or even to southern France. This observation may support the hypothesis that there is gradual northward expansion of *R. sanguineus* (s.l.) within France. There could be emergence of new infestation foci resulting from tick introduction on dogs returning from Mediterranean regions.

Climatic and other environmental factors play crucial roles affecting tick and tick-borne disease distribution [43]. Climate projections for France forecasted an average temperature increase of 1.66 °C (1.41–1.90 °C) by 2020 relative to the 1900–1930 baseline [44]. An intermediate emissions scenario projects further warming of 3.8 °C (2.9–4.8 °C) by 2100, while a very high emissions scenario predicts an increase of up to 6.7 °C (5.2–8.2 °C), significantly exceeding previous estimates from global and regional climate models. These rising temperatures are favorable to some tick genera, and are also expected to result in climatic extremes including floods and droughts, which may be deleterious to ticks. Future climatic changes could therefore contribute to higher or lower tick abundance depending on the species and local conditions. Prolonged tick activity periods are expected for all species, especially if winters are milder, along with further northward expansion of the range of Mediterranean species such as *R. sanguineus* (s.l.).

### Tick-borne pathogens

There are an increasing number of studies aiming to determine human and animal pathogen prevalence in ticks. Results of these studies are not easily compared because there are many critical factors affecting the outcome, including the detection method used, the developmental stages of ticks sample, and geographical tick origin. A study of *I. ricinus* ticks in a recreational forest in Denmark found an infection rate by one pathogen that was 2.7 times higher in adults than nymphs, with a coinfection rate of 12.3% in adult females and 3.5% in nymphs [45].

The prevalence of pathogen DNA varies among tick species tested. In the present study, the highest pathogen prevalence rates were 82.3% in *I. ricinus* and 65.9% in *D. reticulatus*. These two species also had the highest coinfection rates. Up to five different pathogens were identified in a female *I. ricinus* tick. In contrast, *I. hexagonus* and *R. sanguineus* (s.l.) were infected less frequently, although there was a limited sample size for these species.

Among the microorganisms detected by PCR, *Anaplasma* and *Rickettsia* spp bacteria were most prevalent. *Anaplasma* spp. were detected in 75.3% of *I. ricinus* and 45.7% of *D. reticulatus* ticks, while *Rickettsia* spp. were found in 49.5% of *D. reticulatus* and 35.7% of *R. sanguineus* (s.l.) ticks. These results are consistent with previous research, as both *Anaplasma* and *Rickettsia* are known arthropod symbionts, particularly in ticks. Select species within these genera are known pathogens for people and animals, including: *A. phagocytophilum* transmitted by *Ixodes* spp. ticks; *R. slovaca* or *R. raoulti* transmitted by *Dermacentor*; and *R. conorii* transmitted by *Rhipicephalus*.

Pathogen-specific patterns were observed, with higher prevalences of *Borrelia burgdorferi* (s.l.) in *I. ricinus*, *Mycoplasma haemominutum/haematoparvum* in *D. reticulatus*, and *M. turicensis* in *I. hexagonus*. DNA from other bacterial species, including *A. phagocytophilum*, *Bartonella* spp., *Borrelia miyamotoi*, *Ehrlichia* spp., and *Mycoplasma haemofelis/haemocanis*, as well as protozoa such as piroplasms and *Hepatozoon* spp., were detected in a limited number of ticks.

No significant differences in infection prevalence were observed between ticks collected from cats and dogs. Differences in pathogen prevalence between ticks from these host species have occasionally been reported. For instance, a study in Switzerland detected *Rickettsia* spp. more frequently in ticks from cats (40%) compared with ticks from dogs (18%) [46]. A study in Germany reported significantly higher prevalences of *A. phagocytophilum* and *Borrelia* spp. in *I. ricinus* ticks collected from cats compared with ticks from dogs [47].

Lyme borreliosis is a public health threat in Europe and numerous studies have investigated the prevalence of *B. burgdorferi* sensu lato (s.l.) in ticks. Infection rates in *I. ricinus* range from 0% to 50%, with averages of 10–11% in nymphs and 14–18% in adults [48, 49]. In the present study, 10.2% of *I. ricinus* ticks were infected with *B. burgdorferi* (s.l.), a prevalence within the previously reported range (2–22%) for ticks collected from dogs in various European countries [14, 19, 28, 31, 32, 50, 51]. A 14.5% prevalence was reported in *I. ricinus* ticks collected between 2017 and 2024 through the French citizen-science project CiTIQUE [52]. *Borrelia burgdorferi* (s.l.) is a complex of spirochete bacteria comprising 23 genospecies, with 6 confirmed as human pathogens [53]. *Ixodes ricinus* is considered the primary vector for five species: *B. afzelii*, *B. burgdorferi* sensu stricto (s.s.), *B. garinii*, *B. bavariensis*, and *B. spielmanii*, with the first three being the most clinically significant. The present study did not differentiate between genospecies within the *B. burgdorferi* (s.l.) complex. However, other European studies on ticks collected from dogs or cats identified the presence of *B. afzelii*, *B. burgdorferi* s.s., and *B. garinii*, as well as, to a lesser extent, *B. spielmanii*, *B. valaisiana*, and *B. lusitaniae* [16, 19–21, 47, 50]. Human Lyme borreliosis cases have been reported in all regions of France. The eastern regions (Alsace and Lorraine) and the central region (Limousin) are the most affected, whereas the Mediterranean region reports the lowest incidence rates [54]. In the present study, *B. burgdorferi* (s.l.) DNA was detected in a *R. sanguineus* (s.l.) tick collected from a dog near the city of Lyon. A similar case was documented in the UK, where *B. garinii* DNA was detected in a *R. sanguineus* (s.l.) tick from a dog with a history of travel abroad [50].

*Borrelia miyamotoi* is an emerging tick-borne pathogen in the Northern Hemisphere known to cause relapsing fever and is transmitted by the same tick species as *B. burgdorferi* (s.l.). *Borrelia miyamotoi* DNA has been detected in ticks collected from dogs and cats in Germany [47, 51] and Latvia [20]. In France, its presence has been reported in ticks from multiple regions, with a concentration in Alsace [55, 56]. In the present study, the majority of ticks positive for *B. miyamotoi* (10 of 14) were collected from dogs or cats in eastern France.

*Anaplasma phagocytophilum* is the most widespread tick-borne pathogen of veterinary concern [57]. *I. ricinus* is the principal vector in Europe, although other species, including *I. hexagonus*, may be vectors. A recent review of data from 33 countries found that the prevalence of *A. phagocytophilum* in *I. ricinus*varies widely, and commonly ranges between 1.95% and 7.15% [58]. In the present study, *A. phagocytophilum* DNA was detected in only *I. ricinus* ticks, at a prevalence of 1.5%. Higher prevalence, up to 30.9%, is reported in ticks collected from companion animals in other European countries [1, 14, 23, 51, 59]. Marked regional variations were reported, particularly in Belgium and Germany [14, 47]. In the present study, *D. reticulatus* ticks infected with *A. phagocytophilum* were not detected, consistent with previous reports from Austria [60] and France [61].

Hemotropic mycoplasms are small epicellular bacteria that attach to erythrocyte surfaces in infected animals. These microorganisms are responsible for infectious anemia in several mammalian species, including dogs and cats [62]. In dogs, two species have been identified: *Mycoplasma haemocanis* and *M. haematoparvum*, while three species are predominantly reported in cats: *M. haemofelis*, *M. haemominutum*, and *M. turicensis*. Natural transmission routes of canine and feline hemotropic mycoplasms in field conditions remain unclear, and different transmission mechanisms may predominate depending on the host and *Mycoplasma* species [62]. The role of ticks as vectors is not proven. Hemotropic *Mycoplasma* DNA was detected in various tick species, including *R. sanguineus* (s.l.), collected from infested dogs or cats [63–65]. In the present study, DNA from *M. haemominutum/haematoparvum* was frequently detected in *D. reticulatus* (21.8%) and, to a lesser extent, in *R. sanguineus* (s.l.) (10.7%) ticks collected from dogs. Additionally, *M. turicensis* DNA was predominantly detected in *I. hexagonus* (14.3%) collected from cats.

Piroplasms are protozoan blood parasites, including *Babesia*, *Theileria*, and *Cytauxzoon* spp., transmitted to mammals by hard ticks. In the present study, piroplasms were found in 6.4% of *I. ricinus*, 3.6% of *I. hexagonus*, 5.3% of *D. reticulatus*, and 3.6% of *R. sanguineus* (s.l.) tested. Specific PCRs detected *B. canis* in 2.1% of *D. reticulatus* and *B. vulpes* in 0.4% of *I. ricinus*. The few *B*. *canis*-positive ticks were collected from dogs living in southwest France, a region where canine babesiosis cases are reported every year [40]. The prevalence in *D. reticulatus* was lower than reported in other European countries, with 11% of ticks infesting dogs in central Poland [66] and almost 30% in Hungary [36].

The piroplasm *C. felis* is an obligate pathogen of Felids and the agent of cytauxzoonosis, a life-threatening disease of domestic cats in North America. Other species, *C. europaeus*, *C. otrantorum*, and *C. banethi*, have been described in Europe [67]. The tick vectors of European *Cytauxzoon* spp. and their respective lifecycle are not yet known, although *I*. *ricinus* was suggested as a candidate vector [68]. Results of the present study are in accordance with this hypothesis: *Cytauxzoon* DNA was detected in 2.0% of *I. ricinus* and 4.4% of *Ixodes* sp. *Cytauxzoon*-positive ticks were collected almost exclusively from cats and only in northeast France, where wild cats are present.

*Hepatozoon* infection in dogs and cats causes subclinical to severe disease, worsened by coinfection with bacteria or other hemoparasites. Traditionally, *R. sanguineus* is considered to be the only vector for *H. canis* in Europe. However, *Hepatozoon* DNA has been detected in other tick species: *I. ricinus* and *Dermacentor* spp. from various animals (including dogs and cats) in the Netherlands, Croatia, Germany, and Romania, and *I. hexagonus* and *I. canisuga* from foxes in Germany [69–71]. The present study confirmed these findings: *Hepatozoon* DNA was detected in *I. ricinus*, *I. hexagonus*, and *D. reticulatus*, but not in *R. sanguineus* (s.l.). Furthermore, *I. ricinus* was more likely to be infected by *Hepatozoon* spp. than other tick species.

In the present study, DNA from different pathogens was detected directly in ticks, but blood samples from infested dogs and cats were not tested. Pathogen DNA in ticks may originate either from a recent blood meal or from a previously established infection within the tick and it is not possible to differentiate between these scenarios using PCR detection. However, neither dogs nor cats are considered significant reservoir hosts for the majority of pathogens identified in this study, including *Babesia canis* in dogs. The clinical relevance of detecting a pathogen in a tick collected from an animal is unclear and in this study, all dogs and cats appeared clinically healthy at the time of tick collection. Medical records of infested animals could not be reviewed for development of clinical signs suggestive of tick-borne infections. A few studies have examined the relationship between pathogen presence in ticks and the clinical status of the host. In one study, one cat and five dogs parasitized by *A. phagocytophilum*-infected ticks presented with clinical signs including localized swelling or inflammation at the tick attachment site, lameness, or weight loss [14]. Additionally, a dog carrying a tick positive for *Rickettsia* spp. developed arthritis. However, there was no reported significant difference in prevalence of *B. burgdorferi* (s.l.), *A. phagocytophilum*, or *R. helvetica* between ticks collected from animals with clinical signs and ticks from asymptomatic animals.

## Conclusions

Ticks and tick-borne pathogens are year-round risks for dogs and cats across France and the results of the present study support veterinarians’ recommendations for animal owners to be aware of the importance of tick control. Tick species identified were distributed in similar patterns to previous reports from other western European countries, with *I. ricinus* and *D. reticulatus* predmoninant in dogs and *I. ricinus* and *I. hexagonus* predominant in cats. Collected ticks were positive for a variety of tick-borne micro-organism DNA and pathogen prevalence was consistent with ranges reported in previous European studies.

## Data Availability

Data supporting reported results are contained within the article.
